# To drain or not to drain following thyroidectomy. A prospective, randomized study

**DOI:** 10.15537/smj.2023.44.5.20220031

**Published:** 2023-05

**Authors:** Nidhi Mariam George, Tharun Ganapathy Chitrambalam, Pradeep Joshua Christopher, Manish Marlecha, Sundeep Selvamuthukumaran

**Affiliations:** *From the Department of General Surgery, SRM Medical College Hospital and Research Centre, Kanchipuram, India.*

**Keywords:** thyroidectomy, drains, surgery of thyroid

## Abstract

**Objectives:**

To ascertain the use of draining the thyroid bed following surgery.

**Methods:**

Fifty four patients who underwent total thyroidectomy were enrolled in the study between March 2021 and July 2022 and randomly allocated into 2 groups – a drain group and a no drain group. The hospital stay, operating time, post operative pain, post operative complications, cosmesis, and patient’s perspectives were compared.

**Results:**

The mean duration of hospitalization was significantly shorter in the no drain group as compared to the drain group. The post operative pain, as assessed by the Mankoski Pain Scale (MPS) was significantly higher in the drain group than in the no drain group. The cosmetic evaluation undertaken using the Hollander Wound Evaluation Scale, noted that there was a statistically significant difference in scarring between the 2 groups. There was no statistically significant difference in the duration of surgery and post operative complications between the two groups. Patient satisfaction was also noted to be superlative in the no drain group.

**Conclusion:**

The routine drain placement following thyroidectomy places the patient at a disadvantage in terms of longer hospitalisation, increased post operative pain and poor cosmetic outcome.

**T**he field of thyroid surgery has evolved in a circuitous manner over the years. Drains are frequently used after thyroidectomies with the primary aim of halting any potential post-operative bleeding that could obstruct airways and cause respiratory collapse. This bleeding could prove fatal and may necessitate an emergency re-surgery.^1^ This anxiety motivates the surgeon perform routine drainage following any thyroid surgery. However, several studies and meta-analyses have also been unable to exemplify the advantage of drainage in thyroid surgery. In significant post-operative bleeding, drains could clog with blood clots and fail to notify the surgeon.^2^ Factors like patient-, thyroid-, or surgeon-related can prognosticate difficult thyroidectomy. Complications are minimal when thyroidectomy is carried out under ideal circumstances, in a setting of strong anatomical and physiological understanding paired with thorough surgical skills. The surgeon’s skill is of paramount importance in performing a thyroid surgery without complications. Regular drain use cannot be viewed as a replacement for these elements.^3^

While the routine use of drains following thyroidectomy remains a controversial and highly debated topic, the goal of our study was to test the hypothesis that thyroid surgeries performed without the routine usage of drains are more productive than those performed with drains as they decrease the duration of hospital stay, post operative pain and are also associated with better cosmesis.

## Methods

**Figure 1:**
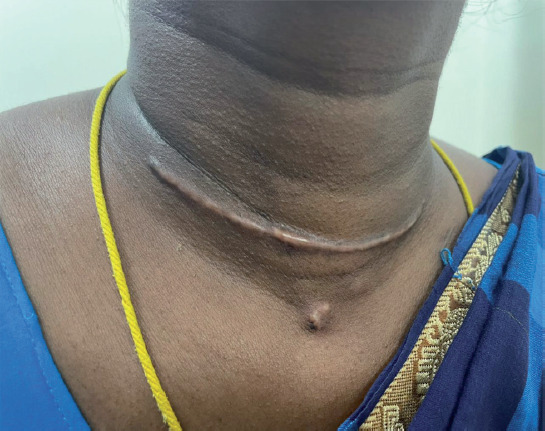
- Post operative image of patient in the drain group.

**Figure 2:**
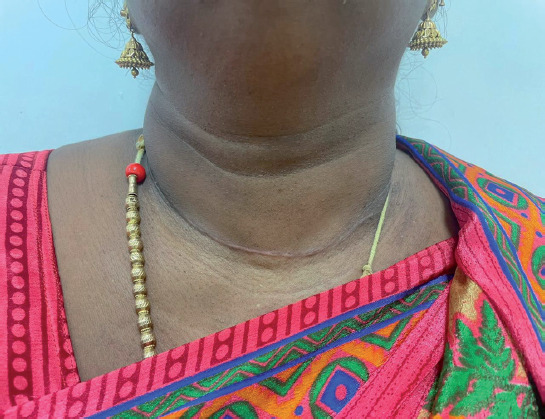
- Post operative image of patient in the no drain group.

A prospective, randomized study was carried out on 54 patients for 18 months from March 2021 to July 2022. We included patients of both genders of all age groups in a euthyroid state with clinically proven and cytologically diagnosed thyroid swelling requiring surgical removal. The exclusion criteria were patients with thyrotoxicosis, on anticoagulants, bleeding diathesis, with huge thyroids more than 6 cm and patients for whom lateral neck dissection was required. They were evaluated using ultrasound neck, thyroid hormone profile (FT3, FT4, TSH), fine needle aspiration cytology (FNAC), Indirect Laryngoscopy and routine pre-operative blood investigations, which included a complete hemogram with coagulation profile, renal and liver function tests, serum electrolytes, serology, urine routine, chest x-ray, neck x-rays, and electrocardiogram (ECG). In addition, the pre-operative evaluation also included 2D echo and pulmonary function tests (PFTs) in applicable patients. Informed consent for surgery and participation in the study was obtained from the patient. A senior surgeon operated on all the patients with strict adherence to intraoperative surgical protocols. Thorough hemostasis and meticulous closure was followed to identify and preserve recurrent laryngeal nerves. Intraoperatively, the patients were allocated using the block randomization method into the drain group (Group A, Figure 1) or the non-drain group (Group, Figure 2). Routine post operative care was administered to all the patients. Post operatively, the patients were evaluated in terms of the duration of their surgery, hospitalization, post operative pain which was recorded using a standard Mankoski Pain Scale (MPS) at 24 hours and 72 hours post operatively. Cosmetic assessment was also carried out using a standard Hollander Wound Evaluation Scale. Finally, the patient’s perspective was also taken into account. The patients who consented to participate in the study were followed up over a period of 6 months.

The study was carried out in accordance with the 1964 Declaration of Helsinki and its subsequent amendments or comparable ethical standards. The protocol of the study was approved by the Institutional Review Board and the Ethics Committee of our hospital.

### Statistical analysis

The data obtained was documented in a standard proforma, analyzed using the Microsoft Excel software and incorporated into Statistical Package for Social Sciences, version 22 (IBM Corp., Armonk, NY, USA). Continuous variables were shown as mean ± standard wherever appropriate. The results were considered as statistically significant when *p*-value of <0.0001.

## Results

**Figure 3:**
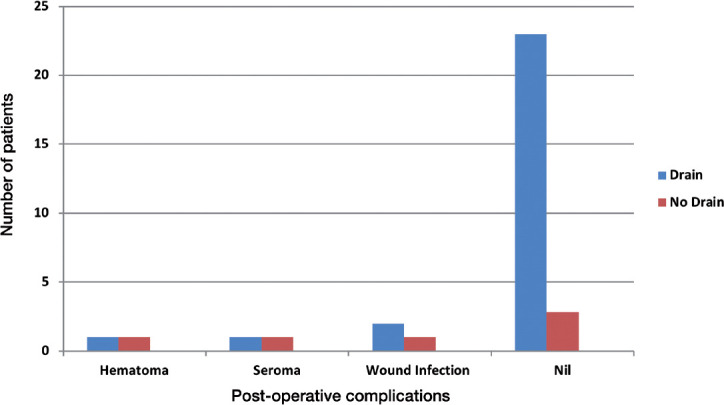
- Post-operative complications among patients with and without post-operative drains following total thyroidectomy.

Based on selection criteria, 54 patients were enrolled in the study of which there was a definite female preponderance at 51 female and 3 male patients who underwent total thyroidectomy. The mean age of the patients was 44.8±6.1 (range 34-57 years). The mean duration of surgery was 68.1 minutes (SD ±9.3) in the drain group, while it was 67.4 minutes (SD ±10) in the non-drain group. There was no significant association between the groups (*p*=0.776). The post-operative pain was assessed 24 hours and 72 hours post-operatively using the MPS. Patients in the drain group had a higher MPS score than those in the no-drain group. The mean MPS of the drain group at 24 hours was 6.0 (SD±1.6) compared to 3.6 (SD±0.9) in the non-drain group. The difference was significant (*p*<0.0001). However, no significant difference in post-operative pain was noted between the groups in the 72 hours of evaluation (*p*=0.366). The main post-operative complications assessed were hematoma, seroma and wound infection as depicted in Figure 3. Patients who developed seromas and hematomas in both the groups were managed conservatively. Postoperative wound infection patients were administered a course of antibiotics. The difference in post-operative complications between the groups was not statistically significant (*p*=0.702).

The patients in whom drains were placed experienced a longer duration of hospitalization (mean 3.0±0.6) as compared to non-drain patients (mean 2.2±0.5). This difference was statistically significant (*p*<0.0001). With the Hollander Wound Evaluation Scale, the drain group registered a mean score of 1.2±0.6, while it was 0.5±0.5 in the non-drain group. The difference in scarring between the groups was statistically significant.

## Discussion

The practise of general surgery has thyroid surgery at its core. Although the customary use of drains following thyroidectomy has been the subject of ongoing dispute, most surgical departments worldwide have a uniform practise of draining the thyroid bed following thyroidectomy. According to prevailing theory, suction drainage limits the build-up of lymphatic and bleeding secretions, decreasing the possibility of neck hematomas and surgical site infections.^4^ However, research has not backed up this theory. Refutations against the routine usage of drains argue that there is increased risk of postoperative infection, cosmetic deficit and prolonged hospital admission outweighing the possible benefits.^5^ Our study aims to establish that the routine use of drains is not only deemed as unnecessary but they prolong the duration of hospitalization, immediate postoperative pain with poor cosmetic outcome. Fifty-four female patients were evaluated for these parameters. In 27 patients, the drain was inserted post-thyroidectomy, while in 27 patients, the drain was not placed.

The study’s results demonstrated that the duration of hospitalization, 24 hours (hrs) post-operative pain, and post-operative wound cosmesis were significantly higher in the drain group compared to the non-drain group. In our research, we discovered that post-operative pain was more common in the drain group, which we hypothesise may be related to the aggravating aspect of having a drain installed. Although the duration of surgery, 72 hrs post-operative pain, post-operative complications of hematoma, seroma and wound infection were higher in the drain group, they did not vary significantly. A key parameter that we have assessed in our study is the cosmetic outcome following surgery. In the present era of minimally invasive and scarless surgery, every surgical wound counts particularly in thyroid disease that predominantly affects females. Drain placement attributes to an additional scar on the exposed part of the neck.

The complication rate following thyroid surgery is very surgeon specific. A meticulously operated thyroid with thorough attention to hemostasis ensures a virtually complication free post operative outcome. A drain does not serve as a proxy for these factors. Sosa et al^6^ carried out a study that established surgeon experience as an important factor in determining the length of stay and complication rate.

The results of our study corroborates with one carried out by Emmi and Reddy^7^ on 44 patients (29 females and 15 males). The average length of hospital stay following thyroid surgery was noticeably longer in the drain arm compared to the no-drain arm. Seven patients in the drain group experienced complications, while just 1 in the no-drain group experienced difficulties. The average length of hospital stay in the drain group was 3.15 days, compared to 2.51 days in the no-drain group. Pain in the drain arm was substantially worse than in the arm without a drain on the first post-operative day (*p*< 0.001). The authors concluded that without drains, thyroidectomy is practicable, less uncomfortable, results in an earlier discharge and hence lower costs, and doesn’t raise the risk of post-operative problems.^7^

**Table 1:** - Comparison of Post-operative wound scarring using the Hollander Wound Evaluation Scale among patients with and without post-operative drains following total thyroidectomy

Group	Mean	Standard deviation	*P*-value
***Hollander wound evaluation scale***			
Drain	1.15	0.60	<0.0001
Non-drain	0.50	0.51	

Portinari and Carcofor^8^ carried out a comprehensive systemic review of 20 randomized controlled trial articles to update the understanding of the drain’s function in thyroid surgery in terms of post-operative complications, namely - re-operation rate for bleeding, progressing to a hematoma, seroma, and wound infection; pain by Visual Analogue Scale at the post-operative day; and duration of hospital stay. Overall, 2,204 patients were studied, of which 1,131 were in the drain group. A total of 1073 in the no-drain group, and clinical outcomes were compared between the 2 arms post-thyroidectomy or lobectomy. Re-operation, hematoma, and seroma were not different between the 2 clusters. Similar to our results, higher surgical pain and longer hospital stays were observed in patients with drains. Patients with drains had a higher wound infection rate; however, it is not statistically significant.^8^

Our study did not reveal any significant benefits in placing drains following thyroid surgery. The hospital stay and postoperative pain was significantly lower for patients without drains. The cosmetic outcome was improved and the patients registered a greater satisfaction level without drain placement.

### Study limitation

The small sample size of our study invariably limits the statistical significance of the results. No significant correlation could be established between the rates of postoperative complications and drains. Larger sample size and multicentric studies can be carried out to further evaluate the necessity of drain placement.

In conclusion, this prospective research was carried out to evaluate, collect further evidence, and gain additional knowledge on the significance of routine usage of the drain. We observed that post-operative pain during the first 24 hours was significantly more common in the drain group. The other significant observations were the drain group’s longer duration of hospitalization and post-operative scarring. Since earlier discharge is made possible for patients without drain, it always reduces the patient’s hospitalization costs. Additionally, patients are extremely concerned about post-surgical scars after any surgical operation. Lesser scarring is associated with better treatment outcomes.
